# The Experiences of Midwives Who Attend Births by Women with Life-Limiting Fetal Conditions (LLFC): A Phenomenological Research Study

**DOI:** 10.3390/healthcare11111540

**Published:** 2023-05-25

**Authors:** Urszula Tataj-Puzyna, Krystyna Heland-Kurzak, Dorota Sys, Beata Szlendak, Maria Ryś, Magdalena Krauze, Barbara Baranowska

**Affiliations:** 1 Department of Midwifery, Centre of Postgraduate Medical Education, 01-004 Warsaw, Poland; urszula.tataj-puzyna@cmkp.edu.pl (U.T.-P.); barbara.baranowska@cmkp.edu.pl (B.B.); 2 Department of Social Pedagogy, The Maria Grzegorzewska University, 02-353 Warsaw, Poland; kheland@aps.edu.pl; 3 Department of Medical Statistics, School of Public Health, Centre of Postgraduate Medical Education, 01-826 Warsaw, Poland; 4 Foundation for Supporting Midwives, 00-112 Warsaw, Poland; szlendakb@vp.pl; 5 Institute of Psychology, Department of Christian Philosophy, Cardinal Stefan Wyszyński University, 01-938 Warsaw, Poland; m.rys@uksw.edu.pl; 6 Department of Obstetrics and Gynecology Didactics, Faculty of Health Sciences, Medical University of Warsaw, 00-581 Warsaw, Poland; magdalena.krauze@wum.edu.pl

**Keywords:** health personnel, fetal diseases, life-limiting fetal conditions, midwifery, stillbirth, perinatal death

## Abstract

Providing care to a woman after a Life-Limiting Fetal Conditions (LLFC) diagnosis is a difficult experience for midwives. This study’s aim is to describe the experience of midwives assisting in births following an LLFC diagnosis. It is a qualitative study using Interpretative Phenomenological Analysis (IPA). Semi-structured in-depth interviews were conducted with 15 midwives with experience in caring for women giving birth following an LLFC diagnosis. The data was analyzed through coding using the MAXQDA tool. The main theme emerging from the experience of midwives concerned difficulty in interacting with the woman giving birth. The analysis singled out four subthemes containing the most significant issues arising from the experience of midwives in caring for a woman giving birth to a lethally ill child: in relation with the woman giving birth; in relation with the child and the family; in relation with oneself; and in relation with the workplace. Midwives should have access not only to solid knowledge about this question, but also to courses developing skills in dealing with difficult situations, in coping with stress, in expressing compassion and, most importantly, in communicating with women and their families in such difficult circumstances.

## 1. Background

The development of prenatal diagnosis has led the fetus being seen as a patient who can receive specialized palliative care from the moment of diagnosis until birth. The pregnant woman who continues with her pregnancy following an LLFC (Life-Limiting Fetal Conditions) diagnosis (in connection with her baby) is also a patient. This particular group of patients requires professional maternity care focused on the life, not the death, of the lethally-ill patient (the fetus) [1].

Obstetric and neonatal teams care for women who give birth to babies with a lethal prognosis. Exact statistics are not available due to difficulties with defining and prenatal diagnosis of fetuses with a potentially lethal prognosis. Data from the European Register deaths from the 20th week on due to congenital anomalies amounts to 42 births out of 100,000, and early neonatal deaths or infant deaths (in the 1st week after birth) amounts to 47 out of 100,000 births, producing a general perinatal mortality incidence of 92 out of 100,000 births (Eurocat website). Other studies suggest figures on the order of 1/11,000 and 2% of all pregnancies in which parents receive diagnoses that limit the life of the developing fetus [2]. In an LLFD situation, we may be dealing with intra-uterine death (IUFD), stillbirth or neonatal death. Clinicians are struggling with the issue of how best to support parents during this usually traumatic experience. Research shows that stillbirth is often accompanied by a deafening silence during which the midwife does not know what to say when handing a stillborn infant or a newborn with abnormalities to its mother [3,4,5].

In their practice, midwives and obstetricians must face various situations involving perinatal loss, intra-uterine fetal death or stillbirth. Caring for families affected by these traumatic circumstances is one of the most difficult experiences for health professionals [6,7,8]. Each type of perinatal death represents a challenge and emotional burden for the health care providers, as each situation is characterized by its own specific circumstances and calls for an individual and culturally differentiated approach to loss and death. Most parents are hopeful that their child will be born alive, notwithstanding the diagnosis with a lethal prognosis [9]. Consequently, a midwife’s repeated exposure to her parturient’s perinatal loss can lead her to feel frustration, helplessness and guilt, and to question her competence [10].

Each family experiences the death of a fetus or newborn infant differently. Upon receiving devastating news that her baby may die, a pregnant woman must go through a birth experience which is most often emotionally arduous for her (and for the baby’s father or support person). Midwives who are directly involved in caring for this group of parents can learn about their needs with sensitivity, and support them in giving birth and saying goodbye to their dying child [5]. 

Studies have shown that a philosophy of care focused on the paradigm of ‘being with’ the woman giving birth and her family can enhance the family’s healing process after the event and strengthen family bonds [11,12]. ‘Midwifery continuity of care’ has also been shown to help in building relationships, resulting from personalized care, trust and empowerment [13,14]. Conducting a birth centered on the midwife’s full involvement in the emotional needs of the woman giving birth leads to an increased sense of fulfillment for the mother, despite the anxiety and pain associated with welcoming and bidding farewell to the newborn child after birth. Studies have shown that such a birth is often accompanied by a deafening silence, during which the midwife does not know what to say as she hands the stillborn child or a newborn with abnormalities to the mother [3,4,5]. For midwives providing care in the birth room, this issue gives rise to anxiety about the parents’ reaction when faced with their stillborn child or a newborn with abnormalities. At the same time, midwives are obliged to provide the best possible midwifery care and, in so doing, must decide on and carry out many issues of a purely organizational nature, including the parents’ viewing and holding the stillborn child and taking photographs. However, there is a lack of clear scientific guidelines concerning proper conduct in such situations, and for this reason the midwives may feel an added burden of responsibility for the quality of care provided [15]. 

There is a lack of qualitative research describing the experiences and emotions of midwives assisting women giving birth with an LLFC diagnosis. The purpose of our study was to learn about and analyze the experiences of midwives assisting in births involving an LLFC diagnosis (intra-uterine death (IUFD), stillbirth or neonatal death). In it, the authors sought to determine how the midwives who take part in such births describe this experience in the context of the difficulties associated with caring for this particular group of birthing women.

## 2. Materials and Methods

### 2.1. Study Design 

Considering the varied emotional reactions of the midwives who form the target group, a methodology was sought that would reflect the subtle differences in the way they communicate their experiences. Interpretive Phenomenological Analysis (IPA) was chosen because it provides the researcher with an opportunity to describe how participants understand their life and the role they play, and how they feel in a particular situation [16]. The main advantage of this method is its ability to examine how specific experiences and events affect a participant’s life. The IPA method uses small population samples and focuses on a deeper analysis of the data reflecting the experience of the study group rather than the population itself. The researcher seeks to get closer to the personal world of the participant, and to adopt his or her ‘inner perspective’ [17,18,19,20]. The IPA method provides an opportunity to learn what significance midwives attribute to their experience of assisting during a birth following an LLFC diagnosis, and makes it possible to analyze and interpret the data thus obtained [21].

### 2.2. Recruitment of Study Participants

Recruiting of midwives began in May 2022 when information about the study was sent by email to 12 hospitals in Poland (with second and third degrees of reference). Eighteen midwives from seven Polish hospitals agreed to participate in the study. Two midwives withdrew from the study before being interviewed, and one interrupted the interview five minutes into it due to the difficult emotions raised by the topic. In the end, 15 midwives took part in the study, after which all had the opportunity to see a psychologist. The participating midwives were provided with thorough information about the study’s objectives and its course. The study was conducted on a voluntary basis, with each person agreeing to take part in the study before it began. The youngest midwife was 30 years old, the oldest was 62, and all had a higher education. In addition, 12 midwives declared that they had completed various specializations. The shortest a participant had worked as a midwife was 7 years, the longest was 38 years. Eleven participants declared that they had personally experienced maternity. Each of the interviewees attended at least five births involving an LLFC diagnosis, with one claiming to have attended 30 such births. The experience of midwives conducting the labor of women with an LLFC diagnosis included stillbirth, intra-uterine fetal death (IUFD) and the live birth of babies who died immediately after birth. The characteristics of the study group are shown in the table below (Table 1).

### 2.3. Interviews and Data Analysis

The interviews were conducted between May 2022 and January 2023. Seven interviews were recorded using the Zoom platform at the participants’ home at the most convenient time for the interviewee. Eight interviews were conducted using a voice recorder during a face-to-face meeting at a location suggested by the midwives. The recordings were conducted by the first author of this article, who is an experienced midwife. The research was based on a pre-arranged set of topics. The preparation of broader research questions made it possible to gather extensive research material. The author’s many years of professional experience allowed her to empathically feel for the world of the participants to the extent possible. The importance of empathy in this type of research is especially important [18]. The partly structured interviews focused on the midwives’ subjective accounts having to do with births following an LLFC diagnosis (Supplementary Materials). When more details were needed to ensure the consistency of the data collected, the interviewees were encouraged by means of questions such as: ‘Can you tell me more about this?’ Individual interviews with each midwife lasted from 45 to 90 min. Following each interview, notes were taken on the observations and reflections of the person conducting the interview. The interviews were digitally recorded, then coded and transcribed. In order to meet IPA standards, the findings were coded by two of the paper’s authors using the MAXQDA software. 

As Smith points out, the IPA standards are strongly idiographic [21]. It was decided, therefore, that the analysis of the interviews would begin with the first midwife. The identification of themes and the ordering was evaluated during team meetings, and only after a consensus was reached the next interview was analyzed. At the end of organizing the statements, a cross-analysis was applied, checking the code table for each midwife’s statements for convergence and divergence. Recurring patterns and emerging issues were identified, creating one primary theme and four subthemes. All co-authors approved the coding tree. The paper was written in accordance with the Standards for Reporting Qualitative Research (SRQR), as stated in the paper. 

## 3. Results

The central part of the analyses focused on the experience of the difficult relation between the midwife and the woman giving birth involving an LLFC diagnosis. In connection with the central theme, four subthemes of analysis of the research material were established: in relation with the woman giving birth; in relation with the child and the family; in relation with oneself; and in relation with the workplace. On the one hand, a key aspect of our approach is the use of the preposition ‘in’ to better reflect the dynamics of events and feeling experienced by the midwives; on the other hand, while analyzing the research material, this allowed us to formulate/establish a specific interpretive argumentation track, which is mandated by the choice of the specific methodological approach (IPA). 

### 3.1. In Relation with the Woman Giving Birth

In the midwives’ descriptions of their experiences, the focus on the woman giving birth comes to the fore. These births were described by the midwives in terms of the needs of the woman in labor, rather than their own experiences. Twelve midwives stated the most common way to cope while conducting a difficult labor was to meet the laboring woman’s expectations about the course of the birth:

‘*For me, the hardest part was to respond to their expectations, because somewhere deep inside I didn’t agree with them. I think that I would have acted differently in their place. But, at the same time I didn’t want to judge them and I wanted to help. So, in order to help them I had to get a feel for how they perceived the situation. And that was probably the hardest thing for me to do. To set aside what I felt in that situation and switch, in a moment, to their point of view*’ (Interview no. 7).

In responding to the expectations of the parturients, midwives need to recognize their needs, and this is difficult in this group of women because it involves asking questions that evoke emotions. Eleven midwives found it stressful to speak with parturients during the first period of labor about various formal issues (such as dealing with contact with the baby after birth, keeping mementos). They were concerned with not burdening the parturient with additional suffering:

*What is probably the most difficult is the first meeting with this woman—when I have to sense what she needs, begin speaking, establish contact. And I don’t know what kind of mental state she is in, whether my questions about listening to her pulse, dressing the baby after birth, saying farewell, etc., will trigger a wave of tears or increase her pain. Whether she will go into a total block, in which I will have no contact with her. Seeking not to add to this woman’s suffering while, and at the same, finding out what her needs are so that I can meet them at least in some small way is very stressful*. (Interview no. 12).

Meeting such expectations was especially difficult in the case of women who avoided contact with medical personnel. The absence of contact and conversation made it difficult to find out what a woman’s needs related to childbirth are: *‘with this patient this was the more difficult [...] as I had the impression that she simply had no need for such contact from me’* (Interview no. 3). 

Establishing a relationship with a woman during labor is also difficult given the strong emotions that arise in both the parturient and the midwife. The tension and anxiety of the parturient are amplified by the midwife’s performance of midwifery procedures:

‘*Listening to the heartbeat, following the progress of labor. I think that, yes, all this is difficult, especially for this woman. But also for me, because she most often takes any new information with anxiety in the 1st period, while in the 2nd period of labor it is the same. When she feels that the resolution is approaching, she tries to suppress it, as if to delay it, to postpone it in time*’ (Interview no. 5).

Five midwives believe that professional experience (birth room staff seniority) can help them establish a relationship with the woman (Interviews nos. 1, 2, 3, 4 or 9). Three note that the peculiarities involved in assisting during a birth of this type do not allow the midwife to prepare for it adequately, especially when the midwife does not know the woman giving birth (Interview nos. 5, 11 or 15).

Midwives held the view that, if possible, physical pain relief should be provided to the parturient during labor. One midwife additionally stressed the importance of an individual approach, even in this question:

‘*It seems to us that the psychological pain is sufficient enough and we wish to relieve the physical pain [...]. But I think that we should still inquire about this, because some people are psychologically structured in such a way that they like to shift psychological pain onto physical pain and somehow this brings them relief, so I am not sure we should treat everyone in the same standard way*’ (Interview no. 4).

The midwives considered it important for parents to say goodbye to their child after death with dignity, and with this in mind, they tried to give the family as much time together as possible: *‘Let’s not take these moments with the child away from them, let them hold it in their arms as long as they need to’* (Interview no. 10).

### 3.2. In Relation with the Child and the Family 

The midwives in charge of the birth tried to do their best to prepare the newborn baby to be shown to the woman and the family. One of the issues identified from the data was the difficulty in dealing with what the baby looked like, as sometimes the newborn was macerated. The midwives were keen to manage the second period of labor in such a way as to cause as little damage as possible to the baby’s delicate tissues. Sometimes they chose to make a perineal incision, mainly so that the baby body would be born with as little damage as possible:

‘*We also try to make sure that this baby suffers as little as possible during the course of the birth, so that visually it shows as little bruising and deformities as possible; so that later, after it has been born and wrapped in tetra cloth or a blanket [...], they can see this child [...] and so that this image, which will remain with them for the rest of their lives, causes as little trauma for them as possible*’ (Interview no. 6).

The statements of the midwives reveal an evident concern to provide a sense of security, for the father/support person who was present at the birth as well. This motivation was inextricably linked to the awareness that this type of event causes major suffering for the family. The midwife’s main tasks:

− to ensure that memories of childbirth are positive (Interview no. 3);
− to make them feel secure (Interview no. 5);
− to support them emotionally (Interview no. 6);
− to spare them additional suffering (Interview no. 7).

An interesting aspect that emerges from this analysis is the account about the baby, in relation to whom all the applicable procedures and solutions are carried out in support of life and health. However, these are births with a lethal prognosis, and it was evident in the midwives’ narratives that in these situations they are primarily concerned for the woman and her well-being. In such a situation, the midwives were aware of their limitations and of the need to live up to their responsibilities at a time when the baby is already dying, or will die soon: *‘We often are aware that this person will be there only for a moment’ (Interview no. 11)*. It seems like the need to accept reality helps the midwives focus on their duties. During such births, the midwives’ attention if most often focused on making the birth as gentle as possible:

‘*they really wonderfully say goodbye to these children and I am full of admiration and very touched, and in part full or joy that we managed to have a wonderful birth in silence in such a marvelous atmosphere*’ (Interview no. 13).

Five midwives who have experience in assisting during births with women who had been prepared, along with their husband/partner, to give birth to a child with a lethal prognosis say that the presence of the child’s father during the birth has therapeutic value:

‘*It seems to me that some couples come to the maternity ward and decide to give birth together without having given it much thought, and they must come to face with various things, while patients who come from the hospice have discussed many things with their partner, with a companion, or with a loved one*’ (Interview no. 11).

In the above scenario, the midwife being interviewed showed the researcher two different perspectives on the conduct of childbirth: the first being when the decision that the father would be present during the birth is taken thoughtlessly and leads to greater hardship during the birth; and the second being when parents duly prepared in a suitable place embark on the birthing process fully aware of what will take place. As they observed the relationship between the parents, eight other surveyed midwives also saw closeness, tenderness and mutual support. Two midwives had experiences of births in which the baby’s father even obstructed the course of labor: *‘This was a really difficult birth mentally, because all the time the father made me responsible for [the actions of] someone else (Interview no. 2), or ‘froze’* (Interview no. 2). 

It should be said that midwives chose different strategies in dealing with women, taking into account firstly the needs of the woman, but also those of the child’s father.

### 3.3. In Relation with Oneself

The midwives’ responses describing their experiences with conducting births of women during whose pregnancy a lethal fetal/baby diagnosis was made contain references to their own emotional difficulties and to their high level of stress and discomfort. Although midwives believe that, in terms of care, these births do not differ much in handling from physiological births, they definitely require a different sort of emotional commitment. As some midwives noted:

‘*Technically, the second stage of birth is really no different than in the case of a woman who gives birth to a healthy baby, because vertical positions are also used. Also, the patient can choose the position in which she is most comfortable*’ (Interview no. 1).

The key to interpreting the midwives’ statements is the word ‘technically.’ For a midwife with many years of experience in the birth room, the management of childbirth, understood as the performance of specific midwifery procedures, is not difficult. As one midwife noted, the difficulty is related to the need for emotional support when providing care at the same time to women giving birth to a healthy and a lethally diagnosed baby:

‘*I know what I can expect when meeting such a patient, any patient, actually. Because, in fact, whether that patient is giving birth to a sick baby, or a stillborn baby, or a normal baby, she pretty much requires the same thing. [...] one speaks one way with a patient who has a physiological pregnancy and a physiological birth, and differently to this [other] type of patient. It is obvious that you have to switch gears here. The worst thing is when one is conducting two births at once, that is, a physiological and a pathological one*.’ (Interview no. 2).

The midwives show that managing two labor processes which take place in neighboring rooms and have different courses and prognoses represents a big challenge in their job, especially when they have to assume the burden of feelings felt as negative by the woman giving birth: *‘I felt blamed [...] with all that happens to me and with my feelings I have to deal myself’* (Interview no. 2). 

These difficult emotions, the stress and discomfort that arise during LLFC births are aggravating, and midwives do not have the space and the time to deal with them. The inability to ‘stop’ for a while and ‘cool off’ is due to the need to go on working on call and caring for other women or to fill out medical forms after the birth:

‘*I will sit down and think for a moment, […], for a minute or two. And I have to deal with the documentation because my shift will end in a moment, or else I have to go see a patient, because just because I assisted during a birth doesn’t mean that I can sit down for an hour, but I need to go see her, to check that she isn’t bleeding, and find out how she feels*’ (Interview no. 2).

Midwives found it emotionally easier to conduct childbirth with women who had prepared for the situation a few weeks earlier. Such preparation was organized by an experienced midwife either at the hospice or during the course of the pregnancy. As the midwives noted, such parents *‘are consciously prepared for the birth of their child. Preparation for a stillbirth is not always possible, however.’* (Interview no. 10).

Most midwives said that it is difficult to care for a parturient who unexpectedly learns about the intra-uterine death of her previously healthy child. Three midwives (Interviews nos. 2, 3 and 15) said that, with this group of parents, the most difficult aspects involve unpredictable reactions in the birth room and communication difficulties. The parents, in their despair, do not want to see the child and blame the midwives for what happened to them. As one midwife related it:

‘*We spend the entire night in the birth room… it is a difficult experience to assist people in this situation... In her despair the mother even denied the humanity of her child and called on us to take “this” away from her…. Having carefully worked with their emotions all night, having overcome this anger, they said farewell to their child; they spend time with it and did everything they possibly could for the child. During the night, they came to terms with being parents to their stillborn child*’ (Interview no. 15).

On the other hand, a loud call for psychological support to be organized for midwives as well emerges from the interviewees’ statements:

‘*I was taught how to receive a birth from a woman who is expecting a healthy baby from the technical point of view, but I was not prepared to receive births from women who give birth to stillborn babies, or babies who will die in their arms. This isn’t easy, no one taught me how to deal with this, I need help to deal with these emotions*’ (Interview no. 12).

The vast majority of midwives participating in the study called for such support. Three midwives shared their experience of seeking psychological support on their own. 

### 3.4. In Relation with the Workplace

Relations with the workplace are understood primarily as ways for the midwife to cope with difficult experiences at work. The most common of these is talking to someone about what took place:

‘*What I need, I think, is to be able to tell someone about it. And that brings relief. And I think, unfortunately I have to admit it, that it’s important that this person be a woman, and it’s wonderful if she is a midwife, that is, someone who walks in the same shoes as me*’ (Interview no. 4).

Such a person has to meet specific requirements; it has to be a trusted person from work, preferably someone with experience in receiving difficult births:

‘*I find it very helpful to talk to someone else, if it happens to be another midwife from the same shift, after work we go for an hour to sit in some quiet place to talk and I tell her about my emotions from the difficult shift*’ (Interview no. 1).

The need to support and exchange experiences with other midwives motivated one midwife to organize periodic meetings at her hospital:

‘*I myself started going to such counseling. Some colleagues from various hospitals and I, five midwives in all, are going to such counseling with a psychologist once a month, so I suggested to the girls that we sit down sometimes, at least once in a while, to discuss our experiences and for each to tell what her approach was because I’m also learning different reactions, so to speak, from them*’ (Interview no. 13).

Eight midwives also shared experiences of creating mutual support groups from the bottom up; most often such support is not formalized. This lack of support procedures may be due to the particularly sensitive subject matter we are dealing with. What was clear from the statements of those interviewed was the need for help from someone who had experienced something similar. Such networks form naturally and bottom-up, as can be seen from the statements of the midwives themselves.

## 4. Discussion

Our research has shown that childbirth following an LLFC diagnosis is a difficult experience not only for the parents, but also for the midwife who conducts them. The main theme that emerged in the midwives’ narratives was difficult relationships. These relationships referred to the woman giving birth, the baby/family, themselves and the work they do.

In this study, we have shown that it was important for the midwives to focus on the expectations of women giving birth to a child with a lethal diagnosis. These expectations concerned the course of birth and, especially, ensuring that it was as gentle as possible. This is consistent with previous studies, which showed that the course of birth and the memories associated with the death of the child long remain in the woman’s memory, and are an important and significant event in her life [22].

Providing empathetic care focused on the needs of a mother of a lethally ill child in labor is emotionally arduous for the midwife. For many years, research has shown that midwives working in the birthing room empathize emotionally with those giving birth [23,24]. The intimate nature of the care they provide causes midwives to experience difficult emotions in connection with the birth and when there is a stillbirth, and caring at once for ‘life and death’. 

Showing empathy and providing good care to their parturients in a way that went beyond the guidelines of standard maternity care was indicated as important by the midwives interviewed. It was also important for them to show a particularly sensitive approach to the family by, for example, extending the mother and father’s stay with their child after death and making a dignified farewell possible. The importance of such an approach is also highlighted in the literature [25].

In our study, the midwives stated that the risk of experiencing dissatisfaction decreases when the mother and father are adequately prepared for the birth. The study shows that in a situation when the child is lost, the midwives and other hospital staff play a role in reducing the affected family’s pain and suffering. This is also borne out by research conducted to date [26]. Previous research shows that a woman and her family are more likely to transfer their suffering and dissatisfaction to the hospital staff in the event of losing their newborn child [26,27,28].

In our study, midwives stressed that the most difficult thing is to assist in a birth when the parturient is surprised by the unexpected situation of stillbirth. The opportunity for the family to prepare in advance and come to terms with the inevitable death of their child leads the parents in the birth room to welcome their child and to celebrate the last moments of their prenatal parenthood in an atmosphere of peace and quiet. The parent’s acceptance of this tragic situation has an impact on the working comfort of the midwives in the birth room and defuses the additional stress load connected with having to deal with the despairing parents’ negative emotions. This is consistent with the research conducted by Denise Côté-Arsenault and Erin Denney-Koelsch, which shows that the parents’ prognostic certainty allows them to take full advantage of the time spent with their child, to prepare for its birth and imminent death and for a life without it [29]. In their study, Lalor et al. stress that the process by which the parents attribute meaning to this negative experience allows them to come to terms with the loss of their child [30].

The midwife is an important member of the medical team and provides direct care to a woman who gives birth to a child with a lethal prognosis. Perinatal palliative care is a challenging experience, as it is a practice in which the emotional support for the woman giving birth and her family is also necessary [31]. Studies conducted to date indicate that midwives/nurses who work with patients who are suffering physically and psychologically can be exhausted by the empathy they show, and this has an impact on the quality of their work. In time, unreleased tensions can lead to continued re-traumatization and increase the risk of professional burnout [10,32,33].

The midwives’ emotional involvement in the parents’ experience of illness, death of the newborn and bereavement can not only contribute to emotional problems, but also to somatic symptoms such as, for example, headaches and stomach aches, palpitations, and sleep problems [23,34,35]. Studies show that midwives have difficulty distancing themselves from the experience of assisting during stillbirth, and that it is often a life changing experience for them [6,35]. Midwives who are exhausted by the emotional compassion they show will later be unable to convey the same authentic compassion towards women under their care [36,37].

In our study, midwives shared various strategies for coping with the experience of perinatal death. Their years of professional experience helped them to establish a relationship with the parturient and conversations with colleagues at work helped them to reduce their level of stress. This is consistent with research conducted by Garcia-Caterina et al., which shows that empathetic listening to the women/families under their care and communicating their feelings to their peers, combined with institutional support, are factors that protect midwives from the negative impact of stressful work [38].

Those midwives who are experienced in conducting births of babies with a lethal prognosis say that those are unique events. Each of these births is different, requires an individual approach and to a certain extent escapes the defining framework of procedures and regulations. To that same extent, knowledge of such births is gained through experience, as senior midwives pass their experience and the ‘culture of practice’ to their younger colleagues [39,40]. On the other hand, a number of studies have proven that midwives’ comprehensive knowledge and experience of attending the birth of a seriously ill or stillborn child, the awareness of which is passed on to the expectant woman prior to the birth of her child, is also important in the subsequent course of mourning in a family [41,42].

### Strengths and Limitations 

Among the strong points of this study is the fact that, as a qualitative study, it has enabled us to learn about the experience of midwives in detail and in-depth. It also made it possible to capture a wide variety of experiences, opinions and beliefs that had previously gone unnoticed in the literature, leading to the uncovering of subtleties and complexities that might have been missed in quantitative studies. Moreover, while admittedly conducting a questionnaire-based interview, the authors were able to adapt their questions depending on the course of the study.

This study’s limitations are largely due to the qualitative nature of the research. The first has to do with the relatively small sample, which meant that the results obtained cannot be generalized. Another one is the selection bias factor—the possibility that the study may only reflect the views of those midwives who, for reasons unknown to the authors, wished to share their experiences and came forward to take part in it, but not of those who did not. Thus, such a sample may not reflect the experiences of the entire population of midwives. Importantly, we do not know the opinions of those midwives who declined to take part in the study or who interrupted the interview on account of their strong emotions connected with this subject. Presumably, their opinions may differ considerably from those of the participating group. The presence of the interviewer may also have influenced the course of the study. 

Ultimately, it should be said that our study is focused on the experiences of midwives who cared for parturients after a prenatal diagnosis with a lethal prognosis for the baby (LLFC). Therefore, it is important to conduct further studies related to loss (miscarriage) and other types of perinatal deaths (unexpected intra-uterine fetal death (IUFD), stillbirth or neonatal death) faced by midwives.

## 5. Conclusions

Our research shows that the level of stress and work comfort experienced by the midwife assisting in the birth of a lethally ill child is related to the quality of preparation of the parturient and the child’s father for birth. There is a need for psychological support for midwives, but also for women facing a lethal prognosis concerning their child [38,43,44,45]. Our study confirms that such preparation and support have a long-term impact. There is no doubt that speaking to a woman about stillbirth can potentially lead to anxiety in the midwife, and the woman giving birth, as well as her family, but this does not mean that these topics should be ignored during prenatal care. It is also worth emphasizing that midwives should be provided not only with a solid knowledge about this question, but also with courses to develop skills in dealing with difficult situations, coping with stress, their ability to express compassion, but most importantly, their ability to talk with women and their families in such a difficult situation.

## Figures and Tables

**Table 1:** Characteristics of the participants.

Person	Age	Personal Exp. of Maternity	Civil Status	Education	Specialization	Years in the Profession	Number of LFD Births	Hospital Location
1	50	Yes	Married	BSc in midwifery	Ob-Gyn and Family	16	10–15	Warsaw
2	36	No	Married	MSc in midwifery	Ob-Gyn and Family	26	10	Warsaw
3	40	Yes	Married	MSc in midwifery	Ob-Gyn and Family	19	10	Warsaw
4	53	No	Married	MSc in obstetricsMSc in family nursing	Ob-Gyn	27	10–15	Warsaw
5	44	Yes	Married	MSc in midwifery	Family	23	10	Warsaw
6	36	Yes	Married	MSc in midwifery	Ob-Gyn	14	10	Warsaw
7	30	No	Married	MSc in midwifery	None	7	5	Warsaw
8	50	Yes	Married	BSc in midwifery	None	34	20	Opole
9	54	No	Married	BSc in midwifery	Ob-Gyn	33	30	Katowice
10	62	Yes	Married	MSc in midwifery	Ob-Gyn	38	20	Łódź
11	37	Yes	Married	MSc in midwifery	Ob-Gyn	14	15	Oleśnica
12	52	Yes	Married	MSc in midwifery	Ob-Gyn	31	10–15	Gdańsk
13	53	Yes	Single	BSc in midwifery	Neonat-Epid	32	15–20	Wrocław
14	41	Yes	Single	BSc in midwifery	None	20	10	Wrocław
15	50	Yes	Married	MSc in midwiferyMA in education	Family	24	5–10	Warsaw

Ob-Gyn—Specialization in obstetrics and gynecological nursing; Family—Specialization in family nursing; Neonat-Epid—Specialization in neonatal and epidemiological nursing.

## Data Availability

The data presented in this study are available on request from the corresponding author.

## Supplementary Materials

The following supporting information can be downloaded at: https://www.mdpi.com/article/10.3390/healthcare11111540/s1, Interview Questionnaire. Questions for Midwives.
