# Supplementary material for: The Experiences of Midwives Who Attend Births by Women with Life-Limiting Fetal Conditions (LLFC): A Phenomenological Research Study
Source: Healthcare (Basel). 2023 May 25;11(11):1540. doi: 10.3390/healthcare11111540 (PMC10252452; doi:10.3390/healthcare11111540)
Supplement: Supplementary file 1 [file healthcare-11-01540-s001.zip › healthcare-2331506-supplementary.pdf]

Interview questionnaire. Questions for midwives

Thank you for taking the time to meet with me to share your experience of managing deliveries with women with an inauspicious prenatal diagnosis.

- **As a midwife, you primarily conduct deliveries with women who deliver healthy babies. Could you tell me about conducting deliveries with women who have had a lethal prognosis for their baby?**
  - Please tell us about a birth with LFD parents that was particularly memorable for you.
  - What is the difference between the births when the baby dies during labor, and those when this happens only a few days after birth?

- **The matter of emotions**
  - What emotions do you feel when you enter the birthing room knowing that the child of the woman giving birth has an inauspicious prenatal diagnosis?
  - What do you most care about when conducting such a birth?
  - How do you see your tasks when conducting such a birth?

- **How does the experience of assisting a woman giving birth to a stillborn child affect your professional and personal life?**
  - Did you ever have a personal experience that helps you in conducting such a birth?
  - Could you tell me more about it?

- **Does conducting such a birth constitute a positive or a negative experience for you? Is it a source of strength or a burden?**
  - Do you see this experience as something valuable in your personal or professional life?
  - Could you tell me more about it?

- **Please tell me about conducting the 1st phase of birth.**
  - How does the 1st phase of birth of such children differ from births of healthy children?
  - What aspect of it is most difficult?

- **Please tell me about the presence of the partner/father during such a birth.**
  - What is the significance of the partner/father's presence by the side of the parturient?
  - Could you tell me more about it?

- **Please tell me about conducting the 2nd phase of birth.**
  - How does the 2nd phase of birth of such children differ from births of healthy children?
  - Could you tell me more about it?

- **Please tell me about the aspect of showing the child to the mother after birth?**
  - What emotions do you feel when you are holding the infant in your hands?
  - What do you see as most important at that moment?
  - Could you tell me more about it?

- **Please compare conducting a birth with a woman who has come to terms with the fact of giving birth to a lethally ill child (through birthing classes or individual meetings with a midwife), and one who hasn't had such preparation.**

- **Please tell me about the rituals used by parents to bid farewell to their child.**
  - Can you tell me more about the farewell rituals used in the birth room?
  - Do you suggest specific forms of farewell to the parents?
  - Do you personally prepare souvenirs for the parents? What emotions do you feel in doing so?
  - Could you tell me more about it?

- **What does verbal and extra-verbal communication with a woman giving birth to a child with lethal defects look like?**
  - Are there communication difficulties with a woman giving birth to a child with a lethal defect?
  - What is more difficult, verbal and extra-verbal communication?
  - Is there a difference between communicating with the mother of an ill child and a healthy one?

- **Please describe the procedures in place at the hospital in connection with such births.**
  - Does the hospital where you work have specific guidelines concerning the conduct of births with women with an inauspicious prenatal diagnosis?

- **Please describe the professional training preparing you to conduct births involving an inauspicious prenatal diagnosis.**
  - Do you feel prepared to conduct births with women with an inauspicious prenatal diagnosis?
  - Where does your knowledge come from?
  - Was there someone who offered you professional assistance?
  - Did you take part in courses or training sessions connected with this subject?

- **Please describe the forms of support the midwife has access to in this a situation.**
  - Do you require support following the experience of assisting a woman during the birth of a stillborn child?
  - Did you obtain, or are you obtaining some form of support?
  - What is your way of recuperation?

- **What is most needed by a midwife who decides to conduct such a birth?**
